# Undifferentiated Nasopharyngeal Carcinoma Presenting with Shoulder Mass

**DOI:** 10.1155/2020/2586248

**Published:** 2020-01-24

**Authors:** Cosphiadi Irawan, Rahmat Cahyanur, Reyhan Eddy Yunus

**Affiliations:** ^1^Division of Hematology-Medical Oncology, Department of Internal Medicine, Faculty of Medicine Universitas Indonesia, Dr. Cipto Mangunkusumo General Hospital, Jakarta, Indonesia; ^2^Department of Radiology, Faculty of Medicine Universitas Indonesia, Dr. Cipto Mangunkusumo General Hospital, Jakarta, Indonesia

## Abstract

Nasopharyngeal cancer (NPC) is the most common cancer among head and neck cancer that usually presented with unilateral neck mass. Unusual symptoms of NPC can lead us to diagnosis misleading and delayed definitive treatment. We present a case of NPC with bone metastasis in the shoulder. A 33-year-old female presented with right shoulder mass caused by undifferentiated carcinoma of unknown primary, based on biopsy of shoulder mass. After four months, she was complaining painless neck swelling, headache, and hearing impairment in the left ear. Bone MRI showed malignant bone tumour in the right humerus. Neck CT scan showed mass in the nasopharyngeal and bilateral lymphadenopathy. Biopsy in nasopharyngeal revealed undifferentiated carcinoma of nasopharyngeal cancer (WHO-3 type A). The patient was diagnosed as NPC stage IVb and thus was treated with palliative chemotherapy. After three cycles of cisplatin docetaxel, patient condition improved.

## 1. Introduction

Nasopharyngeal cancer (NPC) is the most common head and neck cancer. Southeast Asia including Indonesia is the area with high prevalence of NPC cases. Our hospital, as a national referral hospital, had 878 patients with NPC in 2012-2015. The incidence rate for NPC is estimated 6.2/100000 cases yearly [1].

The most common presenting sign of NPC is unilateral neck mass (approximately 80% cases) [2]. A previous study in Cipto Mangunkusumo General Hospital showed neck lump presented in 58.1% [2]. Suzina and Hamzah also reported in Malaysia that unilateral neck mass was the most common presenting symptom (37.5%) [3]. Lee et al. studied 5020 patients in Hong Kong and showed that neck mass was found in 37.3%. Metastasis deposits were only found in 0.3% cases as the presenting symptoms [4].

This article would like to report a rare clinical presentation of NPC that came to medical facilities due to right shoulder mass. Unusual presenting sign leads to misleading diagnosis and delayed definitive treatment. The case that undifferentiated carcinoma of unknown primary based on biopsy result could be end up in NPC, especially in an endemic area, is important to us.

## 2. Case Presentation

A 33-year-old female came into the hospital due to right shoulder mass. She felt increasing pain. She reported that the mass grew rapidly. She consulted the orthopaedic, and biopsy was performed. Biopsy result showed undifferentiated carcinoma of unknown primary site. She had been advised to perform further evaluation, but she preferred complementary and alternative medicine for two months.

**Figure 1:**
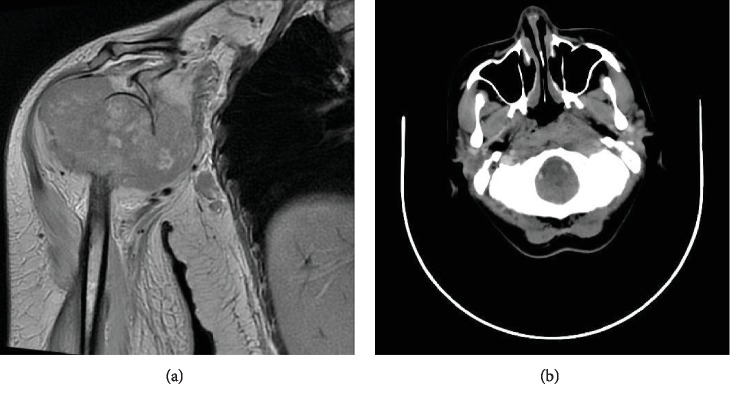
(a) MRI of right upper extremities showed malignant bone tumour. (b) CT scan of nasopharynx showed mass and bilateral lymphadenopathy.

Two months later, she is complaining painless neck swelling rapidly growing in the left neck. She is also complaining headache and hearing impairment in the left ear. She was referred to the Cipto Mangunkusumo General Hospital for further evaluation, because of facility limitation. Bone MRI of right upper extremities was performed and showed malignant bone tumour in the proximal epimetadiaphysis of the right humerus (Figure 1(a)). Neck CT scan was performed and showed mass in the nasopharyngeal and bilateral lymphadenopathy (Figure 1(b)). Biopsy in nasopharyngeal was performed, and undifferentiated carcinoma of nasopharyngeal cancer (WHO-3 type A) was confirmed from histopathological examination.

In the tumour board meeting, after a review with the previous histopathological data from bone and nasopharynx, this case was concluded as NPC stage T4N3M1 (bone). Palliative treatment was performed with Cisplatin-Docetaxel. After three cycles of chemotherapy, patient condition improved, and pain reduced.

## 3. Discussion

Metastasis deposit in the bone, described in this case, was uncommon presenting symptoms for NPC cases (0.3% cases). At the moment of shoulder mass being diagnosed, the patient only had mild headache without other symptoms associated with NPC. Wei and Sham classified common symptoms in NPC into four categories: (1) symptoms caused by the presence of tumour mass in the nasopharynx (epistaxis, nasal block, and discharge); (2) symptoms associated with dysfunction of the Eustachian tube symptoms; (3) symptoms associated with the superior enlargement of the tumour (headache, diplopia, facial pain, and numbness); and (4) neck mass [5]. Among the symptoms that had been classified, neck mass is the most common presenting symptom [4]. The patient was first examined in a health facility with limited equipment, located about 800 km from the capital city. The examination that could be performed to determine the diagnosis was only biopsy of shoulder mass, and the result showed undifferentiated carcinoma of unknown primary. As stated before, there was not any ear, nose, and throat (ENT) symptom when undifferentiated carcinoma of unknown primary was diagnosed so examination leading to ENT disease, such as NPC, was not done there.

This case describes undifferentiated carcinoma found in the bone. The literature stated that 20% of the cases of bone metastasis of unknown primary site were undifferentiated carcinoma. Most of the primary site that caused bone metastasis was lung cancer (52-60%), especially among women and young patients. Besides lung cancer, other origins that are commonly possible were renal carcinoma (12%), prostate cancer (10%), and thyroid carcinoma (3%) [6]. The nasopharynx is an uncommon origin site that caused bone metastasis, without the primary being detected clinically. In spite of this, bone metastasis is the most common location of metastasis in nasopharyngeal carcinoma, ranging from 54 to 80%, with the most frequently involved bone metastatic sites were the rib (53.5%), thoracic vertebrae (50.0%), lumbar vertebrae (43.6%), pelvic girdle (42.3%), and lower limb (32.4%) [7].

A histopathological report in this case showed WHO-3 type A. Undifferentiated carcinoma of nasopharynx characteristic has higher incidence of distant metastasis, despite higher local tumour control rate. Undifferentiated carcinoma is common in an endemic area (account 95% cases), including Indonesia. Type A undifferentiated NPC had a 5-year overall survival (30-40%) [8].

In this case, the patient seek out complementary and alternative treatment before going through further examination. It is quite common in Indonesia to seek out traditional treatment. About 31.4% of the population in Indonesia use traditional treatment [9]. In this case, the diagnosis was delayed and so was the medical treatment. NPC itself has a higher rate to be cured if the patients seek medical treatment in an earlier stage. The 5-year overall survival (OS) rates for patients in stages I, II, III, and IV were 90.48%, 76.71%, 76.89%, and 33.87%, respectively (*P* = 0.000) [10]. There will be more cases found untreatable if there is more delay to be diagnosed and proceed with the medical treatment.

This case had been diagnosed as stage IVb based on the AJCC 8^th^ [11]. Palliative intent chemotherapy was planned. Cisplatin and docetaxel were given in this case. After chemotherapy, clinical and CT scan evaluations were done. The neck mass was reduced partially, shoulder mass disappeared, and patient's complain improved [12].
